# Influence of cardiopulmonary exercise test on platelet function in patients with coronary artery diseases on antiplatelet therapy

**DOI:** 10.1186/s12872-022-02486-z

**Published:** 2022-03-04

**Authors:** Chun Yin, Yanhui Wang, Chunhua Mo, Zong Yue, Yihong Sun, Dayi Hu

**Affiliations:** 1 Department of Cardiology, The First Affiliated Hospital of Chongqing Medical University, No. 1, Youyi Road, Yuzhong District, Chongqing, 400016 China; 2 Department of Cardiology, Chongqing General Hospital, Chongqing, 401147 China; 3 Cardiac Rehabilitation Center, Beijing First Hospital of Integrated Chinese and Western Medicine, Beijing, 100026 China; 4 Department of Cardiology, China-Japan Friendship Hospital, Beijing, 100029 China

**Keywords:** Coronary artery diseases, Exercise test, Platelet function, Cardiac rehabilitation

## Abstract

**Background:**

Cardiac rehabilitation reduces mortality and morbidity rate of patients with coronary artery diseases (CAD); however, acute exercise stimulation may also increase the thrombotic risk through platelet activation. Studies on the effects of cardiac rehabilitation on platelet function have been sparse.

**Methods:**

A total of 28 patients (24 men and 4 women; average age = 54.6 ± 8 years old) with stable CAD were enrolled in this study and divided into Aspirin-treated (n = 11; Aspirin group) and dual-antiplatelet-treated group (DAPT group; n = 17). Symptom-limited cardiopulmonary exercise test (CPET) with a cycle ergometer was performed on all the patients. Before and after CPET, platelet function was evaluated using light transmission aggregometry and whole blood flow cytometry.

**Results:**

All patients completed the CPET without provoked cardiac events, and the mean value of peak oxygen uptake (Peak Vo_2_) was 19.3 ± 3 ml/(kg min). Prior to CPET, platelet aggregation was significantly suppressed in DAPT group compared to Aspirin group (43.0 ± 21.5 vs. 72.9 ± 7.5, *p* < 0.001). CPET promoted platelet aggregation in Aspirin group (72.9 ± 7.5 vs. 80.9 ± 7.6, *p* = 0.005) and DAPT group (43.0 ± 21.5 vs. 50.1 ± 20.9, *p* = 0.010), and platelet count was increased in Aspirin (210.9 ± 54.6 vs. 227.5 ± 58.1, *p* = 0.001) and DAPT group (217.5 ± 63.8 vs. 229.7 ± 63.7, *p* = 0.001). However, the expression levels of CD62p and PAC-1 were not affected by CPET in both groups.

**Conclusion:**

Symptom-limited CPET enhanced platelet aggregation in patients with CAD despite treatment with antiplatelet, mainly via platelet count augmentation, but not through single platelet activation.

*Trial registration*: Effects of high intensity interval training versus moderate intensity continue training in cardiac rehabilitation on platelet function of patients with coronary heart diseases: a exploratory randomized controlled trial.

ChiCTR-INR-17010717. Registered 23 February 2017, https://www.chictr.org.cn/edit.aspx?pid=18206&htm=4.

## Background

Cardiac rehabilitation (CR) reduces mortality and morbidity rate [1]. Life quality of patients with coronary artery diseases (CAD) could be improved by CR [2]. In addition, regular exercises serve essential roles during CR [3]. However, acute and high-intensity exercises may also result in acute myocardial infarction [4] and sudden cardiac death [5]. CAD is a common type of cardiovascular disease, accounting for most exercise-related deaths in people aged over 40, in whom acute coronary artery plaque disruption and thrombotic occlusion were commonly found [6]. Prior to the participation in a CR program, cardiopulmonary exercise test (CPET) was usually used to evaluate the exercise capacity of patients and develop an exercise prescription [7]. This process could raise the concern that acute and sub-maximal/maximal stimulation during CPET may increase thrombotic risk in CAD patients.

Platelets play crucial roles on the pathogenesis of atherosclerotic diseases during the formation of acute thrombus [8]. Activation of the platelet could be involved in exercise-related thrombus formation. However, no definite conclusion could be drawn [9]. Previous studies have revealed controversial roles of exercise on platelet function in CAD patients [10–14], which could be attributed to the non-unified standards across these studies [15]. In previous studies, the intensity of exercise was expressed as work capacity [10, 12] and rate-pressure production [13] during peak exercise stage, and the exercise intensity was not described in one study [11]. As the intensity of acute exercise could affect the platelet function [9], variation in exercise intensity may result in the controversial findings when comparing and analyzing the results in previous studies. Maximal aerobic capacity (Vo_2_ max or Peak Vo_2_) directly measured by CPET is more precise to indicate the limits of the cardiopulmonary system of each CAD patients, and easy to compare among individuals [7], which could be a better marker to elucidate the relation between exercise and platelet function in CAD patients.

In previous studies, numerous methods have been used to evaluate the platelet function: such as the measurement of platelet aggregation (PA) [10, 13, 14], blood serum of *β*-thromboglobulin (*β-*TG) and platelet factor 4 (PF-4) [10], platelet expression of glycoprotein (PAC-1) and P-selectin (CD62p) [13], and platelet function analyzer (PFA-100) [12, 13]. The discrepancy in platelet function test may also contribute to the inconsistent results. The historical gold standard of platelet function test is light transmission aggregometry [15]. Flow cytometry for the evaluation of platelet surface markers is also an accurate method to analyze the activities in individual cells, which is widely used to investigate the effects of antiplatelet drugs [16]. However, limited information was available on the expression profiles of platelet surface markers in CAD patients following acute exercise. In this study, the effects of CPET on platelet function in CAD patients were explored, and the underling mechanisms were also investigated.

## Methods

### Study design

This study was carried out at the Cardiac Rehabilitation Center of Beijing First Hospital of Integrated Chinese and Western Medicine from March until October 2017. Consecutive patients were recruited at the Cardiac Rehabilitation Center and assessed for the suitability for this study prior to their participation in the CR program. The inclusive criteria were as follows: (I) Stable CAD patients with well-documented medical records [17]; (II) At least 4 weeks following acute myocardial infarction; (III) Age range: 18–70 years old. The exclusive criteria were as follows: (I) Left ventricular ejection fraction was less than 40%; (II) Patients with uncontrolled hypertension (> 160/100 mmHg); (III) Individuals treated with anticoagulant; (IV) Patients with other cardiac diseases which are in contradiction to exercise [7]; (V) Individuals with other acute or chronic conditions that may interfere with acute exercise; (VI) Platelet counts were less than 100 × 10^9^/L or more than 350 × 10^9^/L; (VII) Participation in other clinical trials or not willing to participate in the study. The study protocol was approved by the Ethics Committee of Beijing First Hospital of Integrated Chinese and Western Medicine and have been registered on the Chinese Clinical Trail Registry (23/03/2017, No:ChiCTR-INR-17010717). Written consents were obtained from all participants. A total of 28 patients were enrolled, and results of their first CPET were collected for data analysis. According to their antiplatelet treatment plans, the individuals were divided into Aspirin group (Aspirin alone, n = 11) and DAPT group (Aspirin and Clopidogrel, n = 17).

### CPET protocol

No alcohol was consumed and no exercise was carried out by all patients 48 h before CPET. Medication and breakfast were given at least 1 h prior to the test. Symptom-limited CPET were performed between 9 to 11 a.m. using a cycle ergometer (MasterScreen CPX, Jaeger, Germany) according to guidelines’ recommendations [7, 18]. After recovering from pulmonary function test, patients were given a break on ergometer for 3 min, then a 3 min warm up at a speed of 60 cycles/min started with initial power output at 0 W, followed by an equal increase in workload of 15 W every 2 min. Test was terminated when following symptoms or signs were observed: angina, fatigue, ST-segment depression (> 2 mm), reduced systolic blood pressure (> 10 mmHg compared to baseline blood pressure) despite an increase in work load, and other indications according to the guidelines of American Heart Association [19, 20]. A 12-lead electrocardiogram (ECG) was continuously recorded and blood pressure was measured every 2 min during exercise. Data acquisition and analysis were performed using Jlab CardioSoft (version 6.7; Carefusion, USA).

### Blood sampling

On the day of CPET, anti-platelet drugs, which were usually taken before breakfast, were taken after the blood sampling. Ten milliliter blood samples were collected from antecubital vein in sitting position before CPET, and immediately after CPET. The first 2 ml of blood samples were discarded, and the following 2 ml were collected using a tube containing EDTA for complete blood count. The rest were aliquoted into two tubes containing sodium citrate (3.8%), one was used for light transmission aggregometry and the other was subjected to flow cytometry. All samples were stored at room temperature before test and tested within 2 h of sample collection.

### Light transmission aggregometry

Platelet aggregation test was performed using a aggregometer (Agg RAM, Helena Laboratories) as previously described [10]. Briefly, platelet rich plasma (PRP) was obtained by centrifugation of whole blood at 180 g for 20 min, and supernatant was carefully removed and kept at room temperature. Subsequently, the remaining sample was centrifuged at 2000 g (4 °C) for 15 min to isolate platelet poor plasma (PPP). Platelet aggregation was triggered by the addition of ADP (5 µM; Sigma-Aldrich,USA) in aggregation tubes at 37 °C. The results were presented as maximal percentage (%) aggregation.

### Flow cytometry

The expression of platelet surface markers CD62p and PAC-1 were examined using flow cytometry (BD, FACSCanto plus, USA). The protocol has been previously described by Morel et al. [21]. Briefly, a part of whole blood samples were activated by ADP (20 µM) at 37 °C for 10 min. Resting and activated samples were fixed in 1% paraformaldehyde (PFA) at 37 °C for 1 h and stained with the corresponding antibodies: anti-CD61/PerCP, anti-CD62p/PE and anti-PAC-1/FITC (BD,San Diego, CA, USA) in dark at room temperature for 30 min. Then, samples were diluted in PBS until further use. Flow cytometry analysis was performed on 10,000 platelets (CD61/PerCP-positive). The results were presented as percentages of CD62p- and PAC-1-positive platelets in the samples.

### Statistical analysis

Data were presented as mean ± standard deviation and subjected to Anderson–Darling test. For normally distributed data, Student’s t-test was performed. For data that were not normally distributed, Mann–Whitney U test or Wilcoxon signed-rank test was carried out. Categorical variables were summarized using numbers/percentages and compared using Fisher’s exact test. *p* < 0.05 was considered to indicate a statistically significant difference. Data analysis was performed using SPSS (version 18.0; IBM, Chicago, USA).

## Results

### Baseline information and clinical features of patients

All the patients (n = 28) were treated with Aspirin (100 mg per day), and 17 patients were also treated with Clopidogrel (75 mg per day; DAPT group). The baseline information of participants is presented in Table 1. The average age was 54.6 ± 8 years old, and 85.7% of the participants were male. There was no significant difference between Aspirin and DAPT group with respect to age, gender, body mass index (BMI), current tobacco use and alcohol consumption, history of type 2 diabetes mellitus (T2DM) and hypertension (HTN). All participants were treated with Aspirin and Statins, no difference was observed in the treatment of ACEI/ARBs, β-blokers, CCBs and Nitrates between the two groups. Triglyceride (TG) level was higher in Aspirin-treated patients compared to DAPT group (2.4 ± 1.1 vs. 1.3 ± 0.6, *p* = 0.003). No difference was found in fasting blood glucose, hemoglobin A1c (HbA1c), total cholesterol (TC), low-density lipoprotein cholesterol (LDL-c), high-density lipoprotein cholesterol (HDL-c) and left ventricular ejection fraction (LVEF) according to transthoracic echocardiography between the two experimental groups.

**Table 1:** Baseline information of Aspirin treated group and DAPT treated group

	Aspirin (n = 11)	DAPT (n = 17)	*p*
Age, years (SD)	56 (8)	54 (8)	0.494
Male, n (%)	9 (82)	15 (88)	0.999
BMI (SD)	26.9 (2.7)	25.5 (4.3)	0.345
Current smoking	3 (27.3)	2 (11.8)	0.353
Current drinking	2 (18.2)	1 (5.9)	0.543
T2DM, n (%)	3 (27)	6 (35)	0.999
HTN, n (%)	6 (52)	11 (65)	0.701
Drugs, n (%)			
ACEI/ARB	3 27)	6 (35)	0.999
β-blokers	9 (82)	12 (71)	0.668
CCBs	2 (18)	5 (29)	0.668
Nitrates	5 (46)	9 (53)	0.999
HbA1c, % (SD)	6.1 (0.9)	6.2 (0.8)	0.781
Glucose, mmol/l (SD)	5.8 (1.3)	6.3 (1.6)	0.476
TC, mmol/l (SD)	4.3 (0.9)	3.6 (0.9)	0.050
LDL, mmol/l (SD)	2.6 (0.8)	2.1 (0.7)	0.149
HDL, mmol/l (SD)	1.1 (0.4)	1.1 (0.3)	0.991
TG, mmol/l (SD)	2.4 (1.1)	1.3 (0.6)	0.003
LVEF (%), SD	63.6 (7.7)	61.4 (5.8)	0.407

*DAPT* dual-antiplatelet-treated, *BMI* body mass index, *T2DM* type 2 diabetes mellitus, *HTN* hypertension, *TC* total cholesterol, *LDL-c* low–density lipoprotein cholesterol, *HDL-c* high-density lipoprotein cholesterol, *LVEF* left ventricular ejection fraction

### Data of CPET

All the participants completely CPET, and the most common reason for termination was fatigue of lower limbs. Additionally, the tests of three patients were terminated due to ST-segment depression, no acute coronary event or other severe complication was provoked. Furthermore, average exercise duration (warm up and recovery period were not counted) was 6.2 ± 1.3 min; mean value of respiratory exchange rate (RER) was 1.04 ± 0.10; average peak oxygen uptake (Peak.Vo_2_) was 19.3 ± 3 ml/(kg min); the mean value of metabolic equivalent (MET) was 5.5 ± 0.9. There was no significant difference between Aspirin and DAPT group with respect to exercise duration, RER, Peak.Vo_2_ and peak MET (Table 2).

**Table 2:** Results of cardiopulmonary exercise test in Aspirin and DAPT treated group

	Aspirin (n = 11)	DAPT (n = 17)	*p*
HR rest, bpm (SD)	72 (8)	70 (10)	0.655
HR peak, bpm (SD)	116 (18)	116 (17)	0.996
VO_2_ peak, ml/kg (SD)	19.2 (2.3)	19.3 (3.5)	0.948
% VO_2_ pred, % (SD)	71.4 (15.4)	66.9 (12.6)	0.407
Work load max, w (SD)	111.4 (13.3)	109.4 (19)	0.771
MET, met (SD)	5.5 (0.6)	5.5 (1.0)	0.952
Exercise time, s (SD)	6.1 (1.2)	6.3 (1.3)	0.713
RER (SD)	1.06 (0.10)	1.03 (0.10)	0.431

*DAPT* dual-antiplatelet-treated, *HR* heart rate, *VO*_*2*_ oxygen uptake, *MET* metabolic equivalent, *RER* respiratory exchange rate

### Comparison of platelet function at before and after CPET between Aspirin and DAPT group

**Fig. 1:**
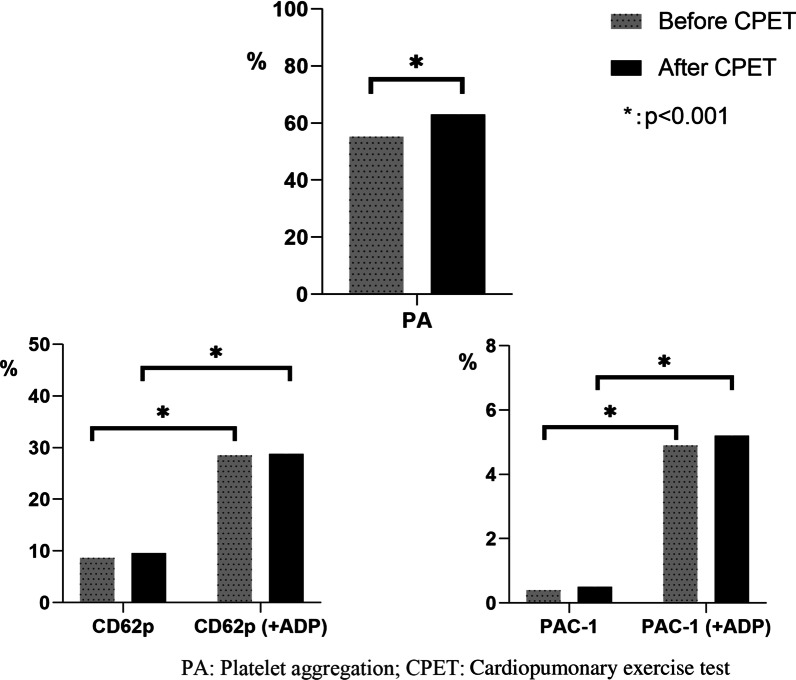
Effects of exercise test on platelet function in total study population

The effects of CPET on platelet function of participants is presented in Fig. 1. Following CPET, platelet aggregation, the levels of CD62p/PAC-1 and ADP-induced expression of CD62p/PAC-1 on platelet surface was significantly enhanced. Before CPET, ADP-induced platelet aggregation was higher in patients treated with Aspirin alone compared to DAPT group (72.9 ± 7.5 vs. 43.0 ± 21.5, *p* < 0.001; Table 3). Platelet count, mean platelet volume (MPV), the levels of CD62p/PAC-1 and ADP-induced expression of CD62p/PAC-1 remained unchanged between Aspirin and DAPT group. Following CPET, platelet aggregation was significantly suppressed in DAPT group compared to Aspirin group (50.1 ± 20.9 vs. 80.9 ± 7.6, *p* < 0.001). Similarly, there was no significant difference between Aspirin and DAPT group with respect to platelet count, MPV, the levels of CD62p/PAC-1 and ADP-induced expression of CD62p/PAC-1 on platelet surface.

**Table 3:** Effects of exercise test on platelet function in Aspirin and DAPT treated group

	Aspirin group (n = 11)			DAPT group (n = 17)		
	Before ET	After ET	*p*	Before ET	After ET	*p*
PA, %	72.9 (7.5)*	80.9 (7.6)**	0.005	43.0 (21.5)*	50.1 (20.9)**	0.010
CD62p, %	8.1 (3.2)	9.7 (5.0)	0.178	6.6 (4.4)	7.3 (3.0)	0.375
CD62p(+ADP), %	33.5 (17.0)	34.5 (12.9)	0.701	22.5 (14.4)	25 (9.4)	0.342
PAC-1, %	0.46 (0.4)	0.57 (0.41)	0.29	0.36 (0.25)	0.51 (0.34)	0.067
PAC-1(+ADP), %	5.62 (2.16)	6.22 (2.88)	0.181	4.4 (1.18)	4.94 (1.7)	0.179
Platelet count, × 10^9^	210.9 (54.6)	227.5 (58.1)	0.001	217.5 (63.8)	229.7 (63.7)	0.001
MPV,fl	10.6 (1.1)	10.7 (0.9)	0.645	11.0 (0.7)	11.1 (0.8)	0.201

*DAPT* dual-antiplatelet-treated, *PA* platelet aggregation, *ET* exercise testing, *MPV* mean platelet volume

*p < 0.001; **p < 0.001

### CPET-induced alteration of platelet function in Aspirin and DAPT group

In Aspirin treated patients, exercise remarkably enhanced platelet aggregation (72.9 ± 7.5 vs. 80.9 ± 7.6, *p* = 0.005) and platelet count (210.9 ± 54.6 vs. 227.5 ± 58.1, *p* = 0.001); however, MPV, the levels of CD62p/PAC-1 and ADP-induced expression of CD62p/PAC-1 remained unchanged (Table 3). In DAPT group, platelet aggregation (43.0 ± 21.5 vs. 50.1 ± 20.9, *p* = 0.010) and platelet count (217.5 ± 63.8 vs. 229.7 ± 63.7, *p* = 0.001) were elevated after exercise; however, there was no significant difference with respect to MPV, the levels of CD62p/PAC-1 and ADP-induced expression of CD62p/PAC-1.

## Discussion

In this controlled clinic trial, the effects of CPET on platelet function in CAD patients were investigated. To our knowledge, this is the first study using CPET to evaluate the influences of acute exercise on platelet function in CAD patients. Our results suggested that ADP-induced platelet aggregation was strengthened by CPET. But these effects could not be suppressed by Aspirin or DAPT. Our results were in consistence with the findings by Brunner et al. [22]. These data indicated that acute exercise can transiently increase thrombotic risk in CAD patients undergoing CPET.

Further experiments were conducted to evaluate the effects of single platelet in response to exercise. Our results revealed that the expression levels of CD62p and PAC-1 remained unchanged in CAD patients following CPET. The expression of CD62p was slightly increased by exercise in both experimental groups, but the difference was not statistically significant. Our results were in consistence with the findings of Kestin et al. [23]. In their study, treadmill test was performed on sedentary individuals until volitional fatigue was achieved without developing the exercise-induced symptoms or coronary insufficiency, but no upregulation of CD62p was induced by exercise. However, as exercise test induced significant ST-depression in CAD patients, enhanced platelet responsiveness to agonist stimulation and increased CD62p expression were detected [24]. Furthermore, remarkable upregulation of CD62p and PAC-1 was observed in healthy volunteers [25, 26] and athletes [27, 28] performing exercise test until exhaustive.

Inconsistency in these results could be caused by different exercise intensities. On the one hand, participants in our study did not reach the exercise intensity to induce significant ST-depression; on the other hand, the mean value of Peak Vo_2_ in the symptom-limited CPET of our study was 19.3 ml/(kg min), which was notably lower compared to the football referees, in whom Peak Vo_2_ was 47.33 ml/(kg min) [28]. The peak RER is used as an accurate and reliable indicator of the effort of patient, and a peak RER of ≥ 1.10 is considered to indicate excellent effort during the test [7]. The average peak RER was 1.04 ± 0.10 in our study, suggesting that the patients achieved a sub-maximal exercise test. Patients with CAD were less likely to achieve certain exercise capacity during the test compared to healthy controls or athletes. This was not only due to physiological/psychological morbidity, but also caused by medical intervention (e.g. treatment with beta-adrenergic receptor blockers), which cannot reach the intensity to result in single platelet activation in CAD patients. As exercise intensity at ventilatory threshold (VT, approximately 45–65% of V_O2_ max) is commonly recommended for exercise training in most CAD patients participating in CR [29], a sub-maximal CPET was relatively safe for CAD patients as single platelet activity was not significantly affected.

Platelet count is not a indicator of platelet activity; however, it can significantly affect the aggregation of platelet [9]. In our study, platelet count was increased by exercise test and platelet aggregation was enhanced simultaneously, while the levels of CD62p, PAC-1 and MPV remained unchanged. Exercise can transiently elevate the concentration of epinephrine, subsequently inducing the release of platelets from the spleen and resulting in platelet count augmentation [30, 31]. Therefore, sub-maximal CPET could promote platelet aggregation in CAD patients mainly via platelet count augmentation, but not through single platelet activation. Anti-platelet therapy plays a central role in secondary prevention of CAD, as robust evidence indicating reduce ischaemic risk in CAD patients. Individualized antiplatelet regimen is beneficial to balance ischemic and bleeding risk in these patients [32, 33]. Antiplatelet drugs are able to inhibit single platelet activity through various signaling pathways; however, they cannot suppress the release of platelets from organs, and this may explain why antiplatelet drugs are not able to inhibit exercise-induced enhancement of platelet aggregation [9, 22].

### Study limitations

In this study, the effects of acute exercise on platelet function were evaluated using CPET and whole blood flow cytometry, and the potential underling mechanisms were also investigated. This data is valuable as the effects of acute exercise on platelet function were quantitatively accessed and further proofed the safety of sub-maximal CPET in CAD patients, and uncovered the mechanism of why antiplatelet drugs are not able to inhibit exercise-induced enhancement of platelet aggregation, which have not been answered in former literature. However, there were still some limitations in the present study: (I) The effects of different gradients of exercise intensity on platelet function were not available and if there was a threshold of intensity above which could lead to significant single platelet activation remained unclear. (II) Analysis of whole blood samples could cause interference of other blood cells with platelets; however, platelet activation could be reduced by using fresh blood samples; (III) This study was not a randomized design and the sample size was relatively small, which should be improved in future.

## Conclusion

Symptom-limited CPET was able to enhance platelet aggregation in patients with stable CAD despite antiplatelet treatment, mainly via platelet count augmentation, but not through single platelet activation. As single platelet function was not significantly affected, a symptom-limited CPET was relatively safe for CAD patients.

## Data Availability

All original data used to support the findings of this study are available from the corresponding author upon reasonable request.
